# Comparison of Locomotor and Feeding Rhythms between Indoor and Outdoor Cats Living in Captivity

**DOI:** 10.3390/ani12182440

**Published:** 2022-09-15

**Authors:** Marine Parker, Jessica Serra, Bertrand L. Deputte, Brunilde Ract-Madoux, Marie Faustin, Etienne Challet

**Affiliations:** 1 Institut des Neurosciences Cellulaires et Intégratives (INCI), CNRS, Université de Strasbourg, 8 Allée du Général Rouvillois, 67000 Strasbourg, France; 2 Royal Canin Research Center, 650 Avenue de la Petite Camargue, 30470 Aimargues, France; 3 Ecole Nationale Vétérinaire d’Alfort (ENVA), 7 Avenue du Général de Gaulle, 94700 Maisons-Alfort, France; 4 Independent Researcher, 92600 Asnières-sur-Seine, France

**Keywords:** cat, housing, feeding pattern, locomotor activity, chronobiology

## Abstract

**Simple Summary:**

Domestic cats are adaptable to various housing conditions, from living in apartments to roaming through hectares of nature. Yet, few studies have been able to determine how the environment they live in affects their daily activity and feeding patterns. To better understand this impact, we used advanced telemetry technologies to compare the daily rhythms of locomotor activity and the feeding of cats living in two housing conditions: an indoor room and a large outdoor enclosure receiving weather fluctuations. Both populations moved and ate more at twilight and when humans were present. Outdoor individuals covered more distance and consumed more food than the indoor ones. However, the indoor cats ate more frequently, were more impacted by human interactions and showed more rhythmic behaviours than outdoor cats, possibly because of fewer distractions. Besides, the outdoor cats were more active at night than the indoor cats, perhaps because their exploratory behaviour is promoted in such environments. This study demonstrates how, on one hand, twilight peaks make the cat still suitable for outdoor life, and on the other hand, integrating human proximity makes it tolerate indoor life. Such observations should be considered in cat housing procedures to better fit to their lifestyle.

**Abstract:**

The plastic nature of cat behaviour allows this “friendly symbiont” of humans to adapt to various housing conditions. Beyond daylight, one could wonder if other environmental factors affect its patterns. Yet, how its activity and feeding rhythms are impacted by its environment is rarely studied in standardised conditions between populations. We compared the behaviour of cats living in a 29 m^2^ indoor room and cats living in a 1145 m^2^ outdoor enclosure, tracking them simultaneously in summer for 21 days, with advanced technologies. Both populations received daylight but weather fluctuations only occurred outdoors. Bimodality was detected in the activity and feeding rhythms of both groups, while twilight triggered crepuscular peaks. Daily, the outdoor population covered more distance (4.29 ± 0.27 km; *p* < 0.001) and consumed more food (67.44 ± 2.65 g; *p* < 0.05) than the indoor population (2.33 ± 0.17 km, 57.75 ± 2.85 g, respectively), but displayed less rhythmic behaviours, assumedly because of rhythm disruptors met only in outdoor conditions. Finally, outdoor housing seemed to promote the exploratory behaviour of the cats at night, while indoor housing increased both meal frequency (*p* = 0.063) and the impact of human interactions on the feeding rhythms of the cats.

## 1. Introduction

The cat is a domestic animal in the sense of being a “friendly symbiont” of humans ([1], as cited by [2]), not as dependent on humans as the dog, for instance. It still constitutes one of the most frequent pets with 15.1 million individuals in France in 2020 [3]. Yet, how its daily activity is affected by different housing conditions, which is of importance for the maintenance of its well-being, is not fully clear. Most chronobiological studies are conducted on laboratory animals to enable proper assessment of rhythms displaying an oscillation of about 24 h, called circadian rhythms (*circa* meaning “around”, *dies* meaning “day”). Indeed, circadian rhythms, controlled by endogenous oscillators, including a master clock located in the hypothalamic suprachiasmatic nucleus (see [4]), and are unambiguously detected when animals are studied in conditions with no synchronization to environmental time cues (called “*zeitgebers*”), i.e., in periods of constant darkness or constant light. In such laboratory conditions, the locomotor activity of the cat has been described as arrhythmic by Hawking et al. [5] and Kavanau [6], but the small sample size (n = 1) and short recording period (eight and seven days in [5] and [4], respectively) make this conclusion doubtful. During more reliable conditions, circadian rhythms were discerned in the cat [7,8], while cats in isolation from humans and human noises exhibited random patterns of activity in constant light, but free-running circadian rhythms in constant darkness [2]. Nevertheless, confinement in cages and controlled conditions in laboratories can erase the effects of external factors determining the natural patterns in animals. Besides, Randall et al. [1] admitted discovering the problem of what the adequate *zeitgeber* in different light/dark cycles is for this species.

From an ecological perspective, the “highly adaptable and opportunistic species” [4] might be predicted to respond with ultradian cycles in a complex environment and exhibit less rigid relation to light/dark transitions. Therefore, in studies conducted in more natural conditions, the question is less about circadian rhythmicity than about nocturnal or diurnal activity patterns. Indeed, the light/dark cycle, recognised as the most potent cue for circadian entrainment in most organisms, can lead to chronotype (i.e., propensity to be more nocturnal or more diurnal) categorisation. Light also suppresses locomotor activity in some mammals (nocturnal species) but promotes it in others (diurnal species [9]). The activity pattern of the cat has most often been described as with a tendency towards nocturnality [7,8,10,11,12,13,14,15,16,17,18]. Other authors detected diurnality; however human interventions or daily feeding conditions were often proposed to be responsible for this timing [5,6,19,20]. In fact, Aschoff ([21], as cited in [22]) rejected nocturnal versus diurnal labels because the same individual may exhibit different patterns on different occasions. Some studies indeed observed either variability among the chronotypes of the different individuals [2,23,24,25] or shifts according to the season [26]. This nocturnal/diurnal distinction may therefore not always be applicable and the undecided literature lets us believe the cat may not fit to it. Be that as it may, crepuscularity seems to best characterise the activity pattern of the cat, with regular peaks occurring near twilight, whether natural or artificial [6,8,11,12,19,20,22,23,26,27,28,29,30,31,32].

There is evidence that different factors such as interaction with competitors and/or with conspecifics, and food distribution interact with photoperiod in the determination of the daily rhythm in the cat. Aside for a high interindividual behavioural variability cited in several studies [2,7,22,29,30,32,33], the behavioural plasticity of the cat is claimed to be responsible for the plurality of findings about its rhythms.

Randall [2,22], referring to the “friendly symbiosis” between cats and humans, pointed out that both food intake and activity in cats are influenced by humans, which can thus be a potent stimulus in determining the pattern of cat activity. High influence of human presence and care on the amount of activity was also reported in pet cats [25].

On another note, the housing conditions of cats may affect their feeding behaviour. Under *ab libitum* diet, cats kept in outdoor individual pens in summer and winter had higher energy requirements than when kept indoors, possibly reflecting the wider ambient temperature range they were exposed to [34]. Housing conditions may also be a significant factor modulating their activity rhythm. Differences in home range size, habitat use and activity patterns between “owned” individuals-i.e., fed and cared by humans and partly living in human dwellings-and “unowned” individuals-i.e., not observed being fed or cared for by humans-were observed on the outskirts of Champaign-Urbana (IL, USA [23]). “Unowned” cats were more active than “owned” cats throughout the year and were more nocturnal in their diel activity, possibly reflecting activity patterns of their prey. Diel activity of “owned” cats, for their part, was more consistent throughout the day, leading the authors to speculate supplemental feeding and the availability of reliable shelter lessens the need for “owned” cats to correspond activity with prey activity patterns. The increased activity in early morning and during evening is considered to come from the availability of the preys in “unowned” cats but from the activity of the owner in “owned” cats. In another study, the activity patterns of one group (B), living in a large house, having free access to a large garden (2000–2500 m^2^) and kept outside at night, was compared to another group (A), which had access to a smaller garden (20–40 m^2^) only during one hour in early morning [25]. Group B was also mainly nocturnal and displaying an active lifestyle, along with presenting a more robust daily rhythmicity than Group A, which was mainly diurnal, and also more active when their owners were home, compared to when they were away. The authors believe restricted activity and exercise area, human care and cleaning can cause shifts in the diurnal/nocturnal active phase and generate weaker rhythms.

However, a longer and continuous recording period (10 days in total or 4 recordings per week in [25] and [23], respectively) and an improved standardisation between the two groups (feeding and living conditions were different between the compared groups for both studies), combined with a feeding behaviour assessment, would allow a better understanding of the housing impact on cats. For this purpose, we investigated factors influencing the daily rhythmicity of locomotor activity and feeding behaviours in cats by using chronobiological tools to compare two populations of cats living in two different housing conditions, but experiencing similar feeding and caring conditions. According to the existing literature, we expect both populations to show rhythm bimodality and crepuscular peaks in their locomotor and feeding patterns. We predict, however, the outdoor population to be less rhythmic in these behaviours, as they live in a more complex and variable environment. On the same note, we hypothesise outdoor individuals to be more active at night than indoor ones.

## 2. Materials and Methods

### 2.1. Animals and Conditions

**Figure 1:**
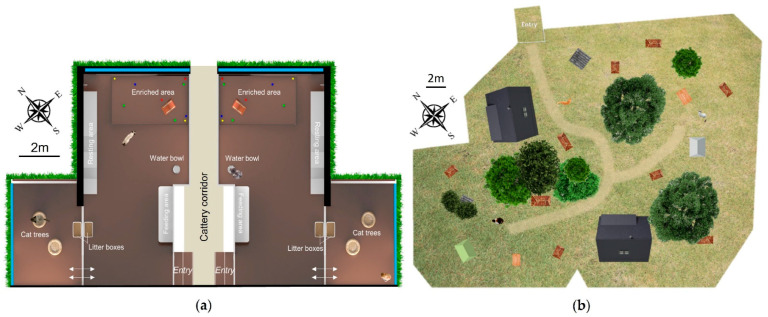
Maps of the (**a**) indoor panels and (**b**) outdoor enclosure.

The indoor population constituted of two groups of neutered domestic cats (*Felis catus*): Group A consisted of six females and two males; Group B of three females and five males. They were aged 1–2 years old and belonged to six different breeds: American shorthair, British longhair, British shorthair, Exotic shorthair, Selkirk rex and Selkirk straight shorthair. The individuals of both groups have been living together for four months prior to the beginning of the study. They were housed at Royal Canin’s cattery located in the South of France (Aimargues, Guard, France). Each group lived in an indoor heated main area of 58.4 m^3^ (L = 6.4 m, l = 3.51 m, H = 2.6 m) and an inner courtyard of 18.1 m^3^ (L = 2.9 m, l = 2.4 m, H = 2.6 m). This courtyard received fresh external air through narrow gaps underneath and on the top of windows, separated from the main area with hermetic cat flaps (Figure 1). In the main room, the cats had free access to a wall shelf containing several hiding places and cushions, an area containing several cat toys, a wall scratching post, a feeding area, a water bowl and a litter tray. In the courtyard, the cats had access to two cat trees containing cushions and another litter tray. According to the population density, the indoor cats had 3.6 m^2^ available per individual.

The outdoor population constituted one group of 27 cats (15 neutered females, 9 castrated males and 3 intact males) aged from 1 to approximately 12 years old, all belonging to European breeds. The cats came from different origins: 20 were previously feral and 7 were previously pet cats, before arriving at the shelter. They had been living together for at least one month prior to the beginning of the study. The individuals were housed in an outdoor enclosure with grass, shrubs and trees, of about 1145 m^2^ at the shelter AVA (Agir pour la Vie Animale) located in the north of France (Cuy-Saint-Fiacre, Normandie, France). Within the enclosure, cats had also access to two chalets containing shelves with cat baskets, nine kennel areas filled with clean straw, two litter areas, two drinking areas containing two stainless-steel water bowls each and two feeding areas, each containing eight doors giving access to two feeding bowls (Figure 1; figures and tables are improved and formatted versions of those used in the PhD thesis [34] during which this study was conducted). According to the population density, the outdoor cats had 42.4 m^2^ available per individual.

**Figure 2:**
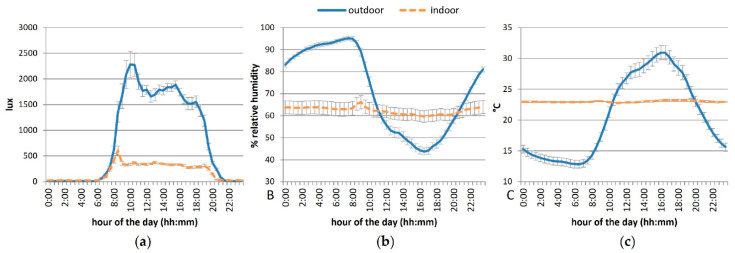
Mean daily luminosity (lux, **a**), ambient temperature (°C, **b**) and relative humidity (%, **c**) according to the housing condition (indoor versus outdoor). Error bars represent standard errors.

The indoor cats were exposed through large windows to natural light/dark cycle, with an average of 14 h 56 min of light per day, daily varying on average from 21–608 lux (Figure 2). Ambient temperature and relative humidity were controlled in the main room, varying on average from 22.8–23.2 °C and from 60–66%, respectively. The outdoor individuals were exposed to climate conditions, receiving on average 15 h 36 min of light per day with ambient temperature daily varying on average from 12.8–30.9 °C, humidity from 44–95% and light from 7–2283 lux (Figure 2).

Because of technical difficulties and unapproachable cats (outdoor), we could not record enough data for every individual. For our analyses, we were able to use the recordings of 15 indoor cats (6 females and 1 male of Group A, 3 females and 5 males of Group B) and only 8 outdoor cats (2 sterilised females, 4 castrated males and 2 intact males). The health of the individuals was regularly monitored via monthly health visits from veterinarians, daily checks from caretakers and regular weighing. This protocol was approved by the ethical committee of Royal Canin and by the ethic regulations of the shelter AVA.

### 2.2. Recording Periods

The locomotor activity and the feeding behaviour of the cats were continuously recorded during 21 days between 11 July and 31 July 2016. The cats were subjected to a week of habituation prior to the 3 week recording period, in order to acclimate themselves to the study conditions (feeding conditions and areas, human interventions, collars).

### 2.3. Feeding and Human Intervention

The cats were fed *ad libitum* with an extruded dry diet (Fit32 (3859 kcal/kg), Royal Canin, Aimargues, France) throughout the study. Food in excess and water were renewed every day at 11:40. As a free-feeding situation, this provided no external constraints on food availability and allowed the animals to choose freely the timing and size of meals.

Human interventions were standardised, i.e., they occurred at the same schedule every day. The animal caregivers entered the panel or enclosure to clean the facilities, interact with the cats and check the good functioning and state of the protocol conditions: between 08:20 and 08:50, between 11:15 and 11:45 and between 15:45 and 16:00 indoors; between 09:15 and 09:30, between 11:15 and 11:45 and between 16:30 and 16:45 outdoors.

### 2.4. Tracking Technologies

Indoors, two small tags were attached to a collar on each cat, one to track their locomotor activity (UWB technology, see [35]), and the second to track their feeding episodes (passive RFID and electronic scales). They continuously provided us with distance covered by each cat every 10 min and the time and weight of consumptions (see [29] for more details). Outdoors, the same tags were incorporated into specifically designed collars to protect them from weather fluctuations. These collars were attached to adjustable break-away collars. A habituation period allowed us to assess that the tags and collars had no adverse effect on the physical condition of the animals and their functioning and correct fitting were checked every day. Preliminary observations were conducted before the study to ensure that the feeding devices did not impact the behaviour of the cats. 

### 2.5. Rhythm Analyses

Using the covered distance and food intake of each cat every 10 min, the same rhythm parameters were calculated and compared as in our previous study [29]. The period (duration of a full cycle, ≈24 h for a circadian rhythm) and amplitude (a measure of how much of the activity or food intake that does occur is actually rhythmic) were measured using the periodogram of the rhythm, calculated by the Clocklab software (v. 2.72, Actimetrics, Wilmette, IL, USA, extension of Matlab v. R2013a, Mathworks, Natick, MA, USA). The Interdaily Stability, Intradaily Variability and onset of the least active 5 h (L5) were calculated by the ActiWatch software (v. 7.31, CamNtech Ltd., Papworth Everard, UK). Interdaily Stability quantifies the degree of resemblance between activity patterns of individual days, Intradaily Variability quantifies the fragmentation of periods of rest and activity and L5 onset yields the time when starts the sequence of the 5 least active hours in the 24-h average activity profile. A strong and robust rhythm was characterised by high amplitude and Interdaily Stability, but low Intradaily Variability. In order to establish if the cats tend to consume/be active more during night or day hours, we assessed their consumption/activity by hour and by day according to the light condition (night hours vs. day hours), using sunrise and sunset hours as phase references. An individual was considered to show a bimodal pattern when the magnitude of the 12 h-peak was equal or superior to half of the 24 h-peak (highest one) on its periodogram. Finally, we used the 10 min periods data to calculate how many times the cats moved and ate in an average day, in both housing conditions.

### 2.6. Statistical Analyses

Every statistical comparison was made with Sigmaplot (v. 13.0, Systat Inc., San Jose, CA, USA). T-tests and Mann-Whitney Rank Sum tests (when the data passed Shapiro-Wilk test for normality) were performed to compare the daily data and rhythmic parameters according to the housing condition (indoor versus outdoor). A one-way ANOVA with repeated measures was performed to compare the distance covered by the outdoor population according to the hour of the day. The distance covered hourly by the indoor population, as well as the hourly food consumption of the two populations not following statistical normality or homogenous variances, Friedman analyses of variances (ANOVAs) with repeated measures were conducted for these data. Two-way ANOVAs were performed to compare the data according to the hour and housing condition and two-way ANOVAs with repeated measures were completed to compare the rhythm parameters according to the variable (activity versus feeding behaviour), as well as the total and hourly covered distance and food consumption according to the light phase. Holm-Sidak post-hoc tests were performed with the ANOVAs and Tukey post-hoc tests with the Friedman ANOVAs. The results are given as mean ± standard error, with a significance threshold of *p* < 0.05 (on graphs: * when 0.01 ≤ *p* < 0.05, ** when 0.001 ≤ *p* < 0.01, *** when *p* < 0.001). Box plots represent first (Q1), second (median) and third quartile (Q3), minimum, and maximum of the data, as well as the smallest data superior to the low frontier (LF = Q1 − 1.5 × (Q3 − Q1)) and the highest data inferior to the high frontier (HF = Q3 + 1.5 − (Q3 − Q1)).

## 3. Results

### 3.1. Locomotor Activity and Behaviour

#### 3.1.1. Locomotor Rhythm

**Figure 3:**
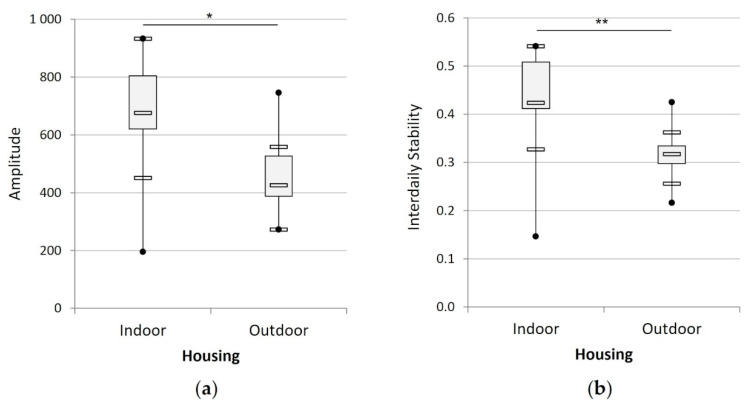
Mean amplitude (**a**) and interdaily stability (**b**) of the activity rhythm of the cats according to their housing condition (indoors (n = 15) versus outdoors (n = 8)). (*) indicates *p* < 0.05, (**) indicates *p* < 0.01.

All the individuals, irrespective of their housing conditions, had 24 h periodicity in their activity rhythm. The activity rhythm amplitude and interdaily stability of the cats were significantly higher indoors than outdoors (t_21_ = −2.83, *p* < 0.05; Mann-Whitney, U = 16.0, *p* < 0.01; respectively, Figure 3).

The intradaily variability of the activity rhythm did not differ significantly between the two populations (1.36 ± 0.05 indoors versus 1.47 ± 0.05 outdoors, t_21_ = 1.42, *p* = 0.172). Many cats showed bimodality in their activity rhythm. Twelve out the 15 cats indoor (80%) and five out eight cats outdoor (63%) displayed a bimodal activity rhythm: the peak of their activity periodogram at 12 h was higher than half of their 24-h peak. The other cats showed unimodal rhythms.

#### 3.1.2. Daily Covered Distance and Frequency of Activity

**Figure 4:**
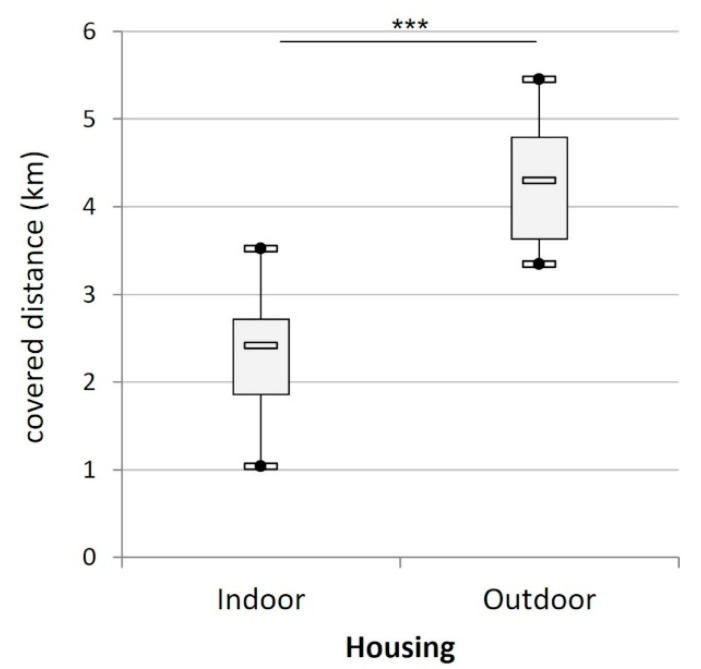
Mean daily distance (km) covered by the cats according to their housing condition (indoors (n = 15) versus outdoors (n = 8)). (***) indicates *p* < 0.001.

The cats covered on average 3.01 ± 0.25 km per day. The outdoor cats covered significantly more distance per day than the indoor cats (t_21_ = 6.33, *p* < 0.001; Figure 4).

The outdoor population tended to move more frequently than the indoor one (108.8 ± 1.5 10-min periods per day versus 101.0 ± 0.6, respectively, t_21_ = −1.906, *p* = 0.070).

#### 3.1.3. Nocturnal versus Diurnal Activity

**Figure 5:**
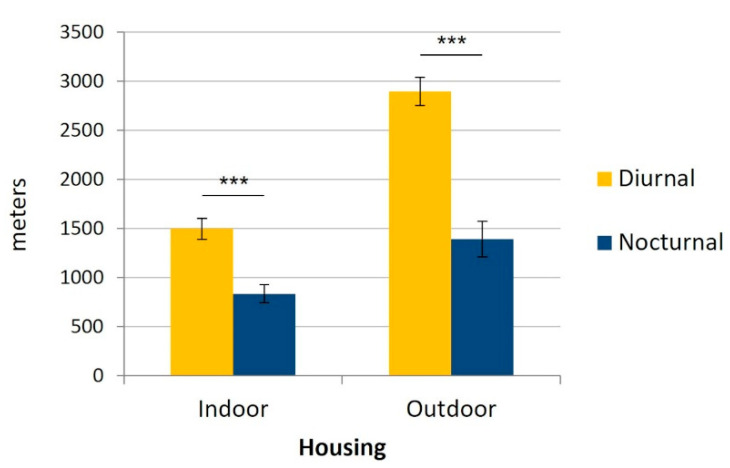
Mean daily nocturnal and diurnal distance (m) covered by the cats according to their housing conditions (indoors (n = 15) versus outdoors (n = 8)). Error bars represent standard errors, (***) indicates *p* < 0.001.

Daily, the cats covered significantly more distance during daytime (1983 ± 165 m) than at night (1028 ± 102 m, *p* < 0.001), regardless of their housing conditions (F_1,21_ = 125.98, *p* < 0.001, Figure 5).

However, the difference between nocturnal and diurnal period was not statistically significant as far as the hourly covered distance is concerned (F_1,21_ = 2.16, *p* = 0.157).

Most of the indoor cats (60%) had their 5 least active hours during the night, whereas the outdoor cats had their 5 least active hours either at midday or at night (Table 1).

#### 3.1.4. Mean Activity Rhythm during the Day

The hour of the day had an impact on the covered distance of the cats (F_23,161_ = 7.05, *p* < 0.001 outdoors; χ^2^_23_ = 246.70, *p* < 0.001 indoors).

Indoors, the cats were more active from the first human intervention to the second (between 08:00 and 12:00), as well as after sunset (between 21:00 and 23:00) and before sunrise (at 05:00; Figure 6a). They covered significantly less distance in the middle of the night (between 01:00 and 04:00) and between the end of human activity in the cattery and sunset (between 17:00 and 19:00).

Outdoors, the cats significantly increased their locomotor activity during human interventions (at 09:00, 11:00 and 16:00, Figure 6b). They were also more active after sunset (at 22:00). Outdoor cats were significantly less active in the middle of the night (between 01:00 and 03:00) and in the middle of the day (at 13:00 and 15:00).

**Figure 7:**
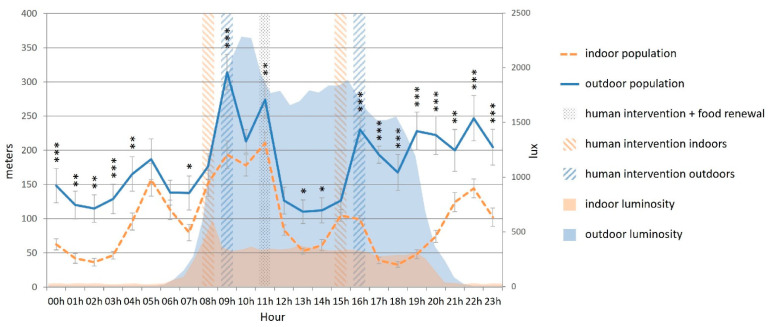
Mean covered distance (m, bars) and luminosity (lux, area) during a day cycle according to the housing condition of the cats (indoors (n = 15) vs. outdoors (n = 8)). Error bars represent standard errors, (*) indicates *p* < 0.05, (**) indicates *p* < 0.01, (***) indicates *p* < 0.001.

The outdoor cats covered significantly more distance than the indoor cats on several occasions during the day and especially during the evening and night (Figure 7).

### 3.2. Feeding Rhythm and Behaviour

#### 3.2.1. Feeding Rhythm

**Figure 8:**
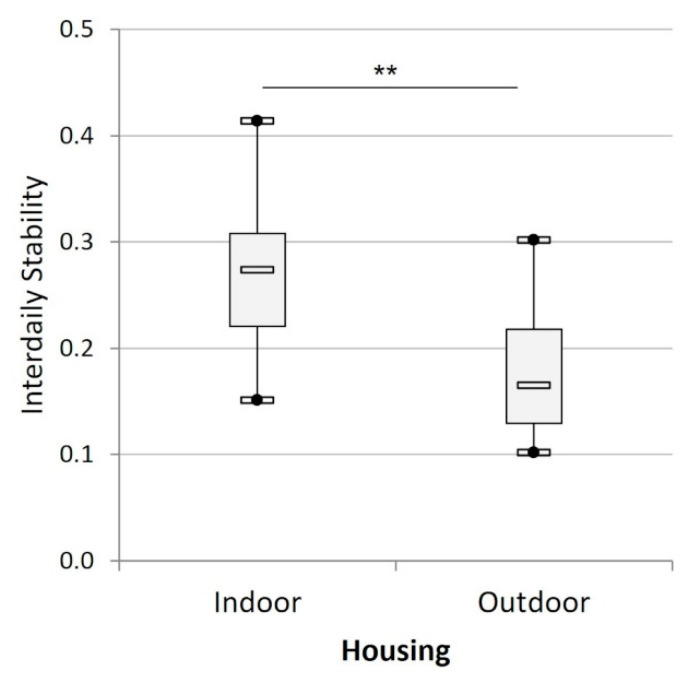
Mean interdaily stability of the feeding rhythm of the cats according to their housing condition (indoors (n = 15) versus outdoors (n = 8)). (**) indicates *p* < 0.01.

All indoor cats and 5 outdoor cats ate with a 24-h cyclicity, the 3 other outdoor cats were arrhythmic. The amplitude of the feeding rhythm of the indoor cats (132 ± 20) did not differ significantly from that of the rhythmic outdoor cats (102 ± 31, t_18_ = 0.76, *p* = 0.457) but the indoor cats showed a significantly higher interdaily stability than the outdoor cats (t_21_ = −2.88, *p* < 0.01, Figure 8).

The intradaily variability of the feeding rhythm did not differ significantly according to the housing condition of the cats (2.09 ± 0.04 indoors versus 2.06 ± 0.06 outdoors, t_21_ = −0.46, *p* = 0.652). 

Most of the indoor cats (12/15, 80%) showed tendencies towards bimodality in their feeding rhythm while the remaining indoor cats displayed unimodal feeding rhythmicity. In contrast, six out of eight outdoor cats (76%) showed either unimodal feeding rhythm (3/8) or arrhythmicity (3/8). Only two outdoor cats (25%) displayed a bimodal feeding rhythmicity.

#### 3.2.2. Daily Food Consumption and Frequency of Food Intake

**Figure 9:**
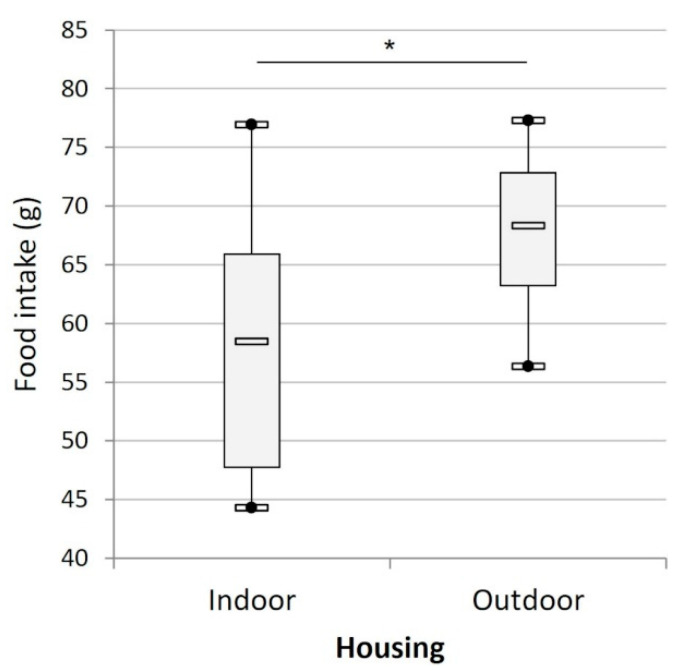
Mean daily food consumption (g) of the cats according to their housing condition (indoors (n = 15) versus outdoors (n = 8)). (*) indicates *p* < 0.05.

The cats ate on average 61.1 ± 2.3 g per day. The outdoor individuals ate significantly more food than the indoor cats (t_21_ = 2.21, *p* < 0.05; Figure 9).

It is worth pointing out that indoor cats only tended to eat more frequently and during a global longer period of time than outdoor individuals (11.5 ± 0.2 10-min periods per day versus 9.3 ± 0.2, respectively; t_21_ = 1.97, *p* = 0.063).

#### 3.2.3. Nocturnal versus Diurnal Consumption

**Figure 10:**
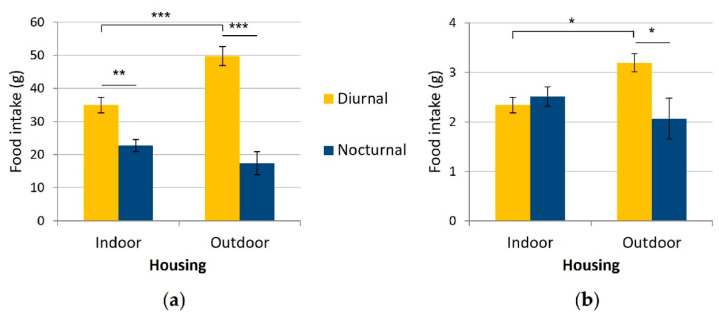
Mean daily (**a**) and hourly (**b**) nocturnal and diurnal food intake (g) of the cats according to their housing condition (indoors (n = 15) versus outdoors (n = 8)). Error bars represent standard errors, (*) indicates *p* < 0.05, (**) indicates *p* < 0.01, (***) indicates *p* < 0.001.

Daily, the cats ate significantly more during daytime (40.1 ± 2.3 g) than at night time (20.9 ± 1.7 g, F_1,21_ = 57.36, *p* < 0.001), be it indoors (Holm-Sidak post-hoc test, *p* < 0.01) or outdoors (Holm-Sidak post-hoc test, *p* < 0.001, Figure 10a). Hourly, food consumption tended to be higher during daytime (2.6 ± 0.1 g per hour) than night time (2.4 ± 0.2 g per hour, F_1,21_ = 3.18, *p* = 0.089, Figure 10b).

There was a significant interaction between housing condition and period of the day in the daily and hourly comparisons (F_1,21_ = 11.73, *p* < 0.01; F_1,21_ = 5.89, *p* < 0.05; respectively): the outdoor group ate significantly more than the indoor group at day/day hours (Holm-Sidak post-hoc test, *p* < 0.001, *p* < 0.05, respectively) but not at night/night hours (Holm-Sidak post-hoc test, *p* = 0.150, *p* = 0.192, respectively).

Also, the more diurnal hourly consumption was significant in the outdoor group (Holm-Sidak post-hoc test, *p* < 0.05) but not indoors (Holm-Sidak post-hoc test, *p* = 0.591).

The indoor cats (60%) had their 5 least active hours either during the night (47%) or in the middle of the day (33%), whereas most of the outdoor cats had their 5 least active hours at night (Table 2).

#### 3.2.4. Mean Feeding Rhythm during the Day

The hour of the day also had an impact on the food intake of the cats (χ^2^_23_ = 57.31, *p* < 0.001 outdoors; χ^2^_23_ = 183.72, *p* < 0.001 indoors).

**Figure 11:**
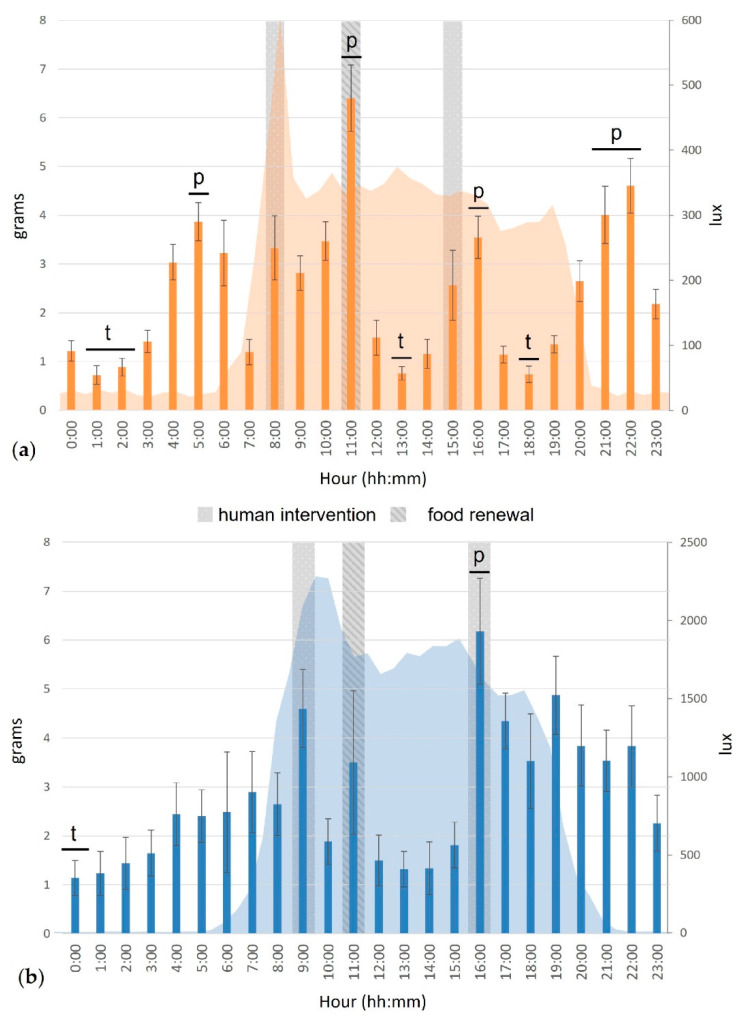
Mean food consumption (g, bars) and luminosity (lux, area) during a day cycle of the cats, indoors (**a**, n = 15) and outdoors (**b**, n = 8). Error bars represent standard errors, (p) indicates a significant peak, (t) indicates a significant trough (for more details, see [29]).

The indoor cats ate significantly more at 05:00 (before sunrise), 11:00 (when food was renewed and humans interacted with the cats), 16:00 (after the last human intervention) and between 21:00 and 23:00 (after sunset, Figure 11). The cats ate significantly less between 01:00 and 03:00 (in the middle of the night), at 13:00 (middle of the day) and 18:00 (before sunset). 

The food intake of the outdoor cats was significantly higher at 16:00, during the last human intervention in the enclosure, and significantly lower at 00:00 (Figure 11).

**Figure 12:**
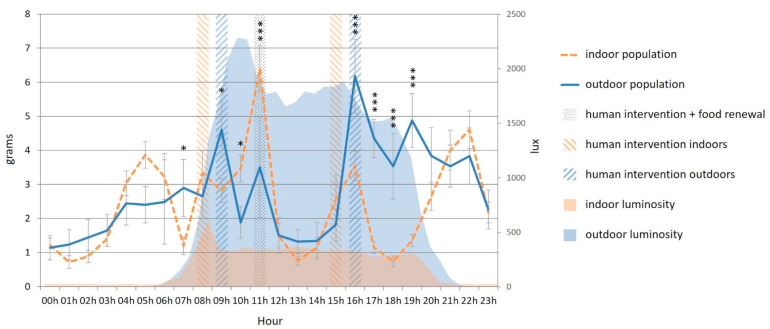
Mean food consumption (g, every hour) of the cats throughout the day according to their housing condition (indoors (n = 15) versus outdoors (n = 8)). Error bars represent standard errors, (*) indicates *p* < 0.05, (***) indicates *p* < 0.001.

The outdoor cats ate significantly more than the indoor cats at 07:00, 09:00 (during the first outdoor human intervention) and from 16:00–20:00 (Figure 12). The indoor cats ate significantly more than the outdoor cats at 10:00 and at 11:00, when food was renewed (Figure 12). 

### 3.3. Eating versus Locomotor Activity Rhythm

The amplitude and interdaily stability of the locomotor activity rhythm were significantly higher than those of the feeding rhythm of the cats (600 ± 41 versus 112 ± 16, F_1,21_ = 199.90, *p* < 0.001; 0.39 ± 0.02 versus 0.24 ± 0.02, F_1,21_ = 69.39, *p* < 0.001; respectively; Table 3) and the intradaily variability of the locomotor activity rhythm was significantly lower than the intradaily variability of the feeding rhythm (1.40 ± 0.04 versus 2.08 ± 0.03, respectively, F_1,21_ = 257.62, *p* < 0.001), be that indoors and outdoors (Holm-Sidak post-hoc test, *p* < 0.001 for all).

## 4. Discussion

This study enabled us to compare the feeding and locomotor activity rhythms of cats according to their housing condition, one population living in an outside enclosure of about 1100 m^2^, the other in indoor panels of about 30 m^2^ each. Although our study conditions prevent the assessment of circadian rhythms in our cats (i.e., no recordings of free-running behaviour in constant darkness or constant light conditions), we were able to determine that a great majority of cats moved and ate with 24-h cyclicity, thus following day-length periodicity. The arrhythmicity in the eating behaviour of three outdoor individuals must be put in parallel with the weaker rhythms we detected in this housing condition. Lessened rhythmicity in cats living in larger outdoor environment differs from the findings of Piccione et al. [25] who observed a more robust daily rhythmicity in cats having *ad libitum* access to a large outdoor garden compared to cats having access to a small one, one hour a day. This could be due to unstandardised conditions in the study of Piccione et al. [25] where owners interacted at will with their pets when at home. Moreover, the indoor population of our study received little disturbance in their daily activity, apart from the three daily standardised human interventions, human noise in the cattery, behaviour of their conspecifics, and the weather fluctuations on the other side of the windows; whereas, in addition to what received the indoor cats, the outdoor population was exposed to direct weather fluctuations, a larger number of conspecifics with varied experience and even occasional fauna living in the enclosure. All these stimuli may constitute rhythm disruptors outdoors that were not met indoors.

On a daily basis, the outdoor population covered more distance than the indoor population, as observed in Horn et al. [23] and Piccione et al. [25]. Knowing that this population was housed in an enclosure about 39 times larger than the indoor panels and that feral cats and free-roaming pet cats can cover up to 1.7 km between diurnal locations [12] and 2.3 km from their home [36], respectively, such results were expected. Furthermore, the activity of these predators may be enhanced by an outdoor environment large enough to run through it and giving access to numerous stimuli such as tall grass, insects, and even small rodents or birds, which can also explain the tendency of the outdoor cats to move more frequently than the indoor cats. On another note, both populations covered more distance during the photophase than during the scotophase. The long photoperiod met at this season (around 15 h indoors and 15 h 30 min outdoors) could be responsible for this finding as the difference is not detected when looking at the hourly covered distance. Our cats were thus not necessarily more active at day hours than at night hours, despite diurnal human impact, possibly because of nocturnal activity outdoors and diurnal activity troughs indoors.

As observed in previous studies [22,25,29], the activity of the cats increased during human presence. They were also more active when sunset ended and prior to sunrise indoors, reminding the findings of many studies where peaks of activity rose around twilight [6,8,11,12,19,20,22,23,26,27,28,29,30,31,32]. Relative troughs in activity were detected in the middle of the night and in the middle of the day for both populations. This echoes with our previous findings in similar indoor conditions [29,30] as well as with lowest activity or observability of cats at midday in two studies on the activity of cats during day hours [28,37]. Rhythm bimodality is therefore met in both populations.

The main difference between the daily activity patterns of the two populations resides in the evening and at night; outdoor cats were significantly more active than the indoor group, whereas differences between the two populations were less pronounced from morning to end of afternoon. This reminds of the higher levels and more prolonged nocturnal activity in the “unowned” cats [23] and cats having access to an outdoor garden [25]. In fact, the outdoor group stayed active after the afternoon human impact, whereas the indoor group seemed to show a longer midday activity trough, with low activity from 12:00–20:00 except at moments when humans enhanced it. This could represent a preservation of nocturnal exploratory behaviour when cats live in an outdoor environment. In addition, the activity intensified during the morning when human intervention was more pronounced outdoors, possibly on the grounds that the cleaning staff did not interact with the indoor cats at 08:00, whereas interactions happened outdoors at 09:00.

In accordance with the results of daily locomotor activity, the daily food intake of the cats was larger outdoors (around 260 kcal ingested per day), where energy requirements should be higher, than indoors (around 223 kcal ingested per day). Nevertheless, confirming previous results mentioned in the introduction [34], the indoor individuals tended to eat more frequently than the outdoor ones. Once again, the more confined area that the indoor population lived in may be the reason of this tendency; the feeding area being closer to the cats during the day—i.e., more easily accessible—and distractions being less frequent, the cats may eat more often indoors than in a large outdoor enclosure to palliate possible boredom.

Crepuscular peaks of food intake rose indoors around twilight, as previously observed in Parker et al. [29,30]. In accordance with the literature [2,7,29,30], humans induced consumption in the cat, resulting in higher diurnal than nocturnal food intake. Besides, food intake of the indoor population was most affected by humans and food renewal at 11:00. More pronounced human impact indoors could be predicted, as the indoor individuals were more familiar and interacting with the caregivers than the outdoor ones. In addition, humans represented a more accessible stimulus in about 30 m^2^ than in about 1100 m^2^. This could also result from longer time necessary outdoors to renew the food, compared to indoors where humans were thus available longer. Only one consumption peak was significant in the outdoor daily pattern, during the last hour humans were present in the enclosure. The outdoor cats are therefore more impacted by humans in their locomotor than eating behaviour. Furthermore, the less rhythmic feeding pattern outdoors may explain the lack of significance in the consumption peaks and troughs in this population. Still, as found in our previous studies [29,30], the least active 5 h (L5) of food intake indicated the cats ate the least in the middle of the day and in the middle of the night in both populations.

The moments outdoor cats ate more than indoor cats reside mainly at 09:00 when humans entered the outdoor enclosure for the first time of the day and more interestingly at the end of the day, between 16:00 and 20:00. During this end of the afternoon, the outdoor population did not diminish their consumption after the last human-induced peak of the day, contrary to the indoor population. In accordance with these observations, evening feeding L5 are never found outdoors and, indoors, coincide with the activity trough occurring at the same time. This comparison reminds us of the difference in activity patterns in the evening and night between the two populations, although this time, the feeding pattern contrast is shorter in time, as the cats recover similar rhythm from 20:00. It thus seems cats are prone to exploratory behaviour, more than eating behaviour, in outdoor nocturnal environments.

Once again [8,22,29,30,38], bimodality, indicated by the periodograms, is an important characteristic of the activity and feeding pattern of the cats and does not vary markedly according to the housing condition. The locomotor activity behaviour of the cats is systematically more rhythmic than their eating behaviour [29,30]. In addition, to corroborate with Johnson et al. [7], Randall et al. [2,22], Refinetti et al. [38], Thorne [39], Zhang et al. [32] and our previous results [29,30], variability is detected among the individuals of a same group—as demonstrated with variable patterns (variable L5 for example)— although some aspects are common among individuals, such as crepuscular and human influence.

This study focused on the impact of housing conditions on the locomotor and feeding behaviour of cats. Some other factors, such as body weight [40], sex [41,42] or sexual status [41,43] of the cats, have been reported to influence their use of space. Due to sample size limitations in our populations, we were unable to test the effect of these factors on the locomotor and feeding behaviours of the cats. In future analyses, considering the different characteristics of the individuals would shed interesting and more detailed insight on the variety of behavioural patterns. It is also worth mentioning that the sample sizes were different between indoor and outdoor populations, essentially due to the difficulty of controlling samples in shelters. Keeping this bias in mind, further experiments using larger and identical sample sizes will be needed to confirm the present findings. Furthermore, as some seasonal variations have also been reported in the activity and feeding behaviours of cats [30,44], the inclusion of all seasons would allow generalising their behaviour on an annual basis.

Although the populations (mainly neutered individuals) and study sites (closed enclosure/rooms) prevent the generalisation of our findings to overall cat populations, this study concerns a significant part of them and provides a design better suited for scientific analyses. Nowadays, more and more pet cats are living in captivity with reduced access to the outdoors, especially in areas where their impact on surrounding wildlife is becoming problematic [45].

## 5. Conclusions

On one hand, similarities are observed between the two populations. Human impact is systematic and even seems to result in more diurnal eating behaviour. Twilight contributes to crepuscular activity and eating peaks and both indoor and outdoor cats decrease activity and food intake in the middle of the day and early afternoon. On the other hand, differences according to the housing condition are flagrant. In their larger enclosure, the outdoor cats cover more distance, feed more and are more active than the indoor population mainly in the evening and night hours, reflecting nocturnal exploratory behaviour. They are also less rhythmic than the indoor cats, supposedly because of more rhythm disruptors in their environment. The indoor individuals, receiving less environmental stimulation, may on their part inhibit nocturnal behaviour and develop a more rhythmic routine with higher human impact on their eating behaviour and more frequent meals. Such observations should be considered in cat housing procedures in order to better fit to their lifestyle.

To conclude, domestic cats enjoy great flexibility in their behaviour. Despite domestication, they stay adapted to life outdoors by preserving ancestral traits of an outdoor predator—as visible in crepuscular peaks maintained indoors. Still, they also fit into indoor life, integrating human proximity in their everyday life. This explains the multiplicity and durability of cat populations on earth, ranging from pet cats living in apartments to feral cats roaming through hectares of nature.

## Figures and Tables

**Figure 6:**
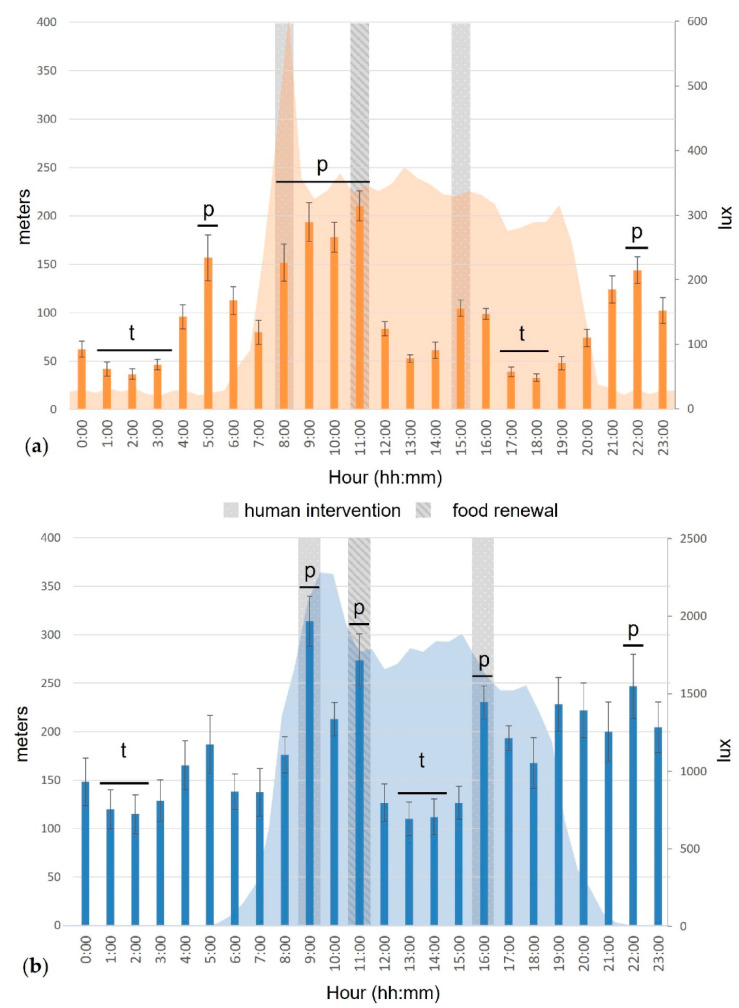
Mean covered distance (m, bars) and luminosity (lux, area) during a day cycle of the cats, indoors (**a**, n = 15) and outdoors (**b**, n = 8). Error bars represent standard errors, (p) indicates a significant peak, (t) indicates a significant trough (for more details, see [29]).

**Table 1:** Time of onset of the Least active 5 h of the cats according to their housing condition. (+) represents one cat.

Housing Condition	Onset of the Least Active 5 h	n
Indoors (n = 15)	Middle day (12:00–14:00)	++
	Afternoon (16:00–17:00)	++++
	Night (23:00–1:00)	+++++++++
Outdoors (n = 8)	Middle day (12:00–14:00)	++++
	Afternoon (16:00–17:00)	0
	Night (23:00–1:00)	++++

**Table 2:** Time of onset of the Least active feeding 5 h of the cats according to their housing condition. (+) represents one cat.

Housing Condition	Onset of the Least Active Feeding 5 h	n
Indoors (n = 15)	Morning (05:00–06:00)	+
	Middle day (11:00–12:00)	+++++
	Afternoon (17:00)	++
	Night (23:00–2:00)	+++++++
Outdoors (n = 8)	Morning (05:00–06:00)	++
	Middle day (11:00–12:00)	+
	Afternoon (17:00)	0
	Night (23:00–2:00)	+++++

**Table 3:** Mean ± SE of amplitude, interdaily stability and intradaily variability according to the rhythm (locomotor versus feeding) and the housing condition of the cats.

Housing Condition	Rhythm	Amplitude	Interdaily Stability	Intradaily Variability
Indoors (n = 15)	Locomotor	674 ± 47	0.43 ± 0.023	1.36 ± 0.05
	Feeding	132 ± 20	0.27 ± 0.02	2.09 ± 0.04
Outdoors (n = 8)	Locomotor	461 ± 52	0.32 ± 0.02	1.47 ± 0.05
	Feeding	75 ± 23	0.18 ± 0.03	2.06 ± 0.06

## Data Availability

Restrictions apply to the availability of these data. Data was obtained from Royal Canin and are possibly available from the authors with the permission of Royal Canin director of laboratory.
